# Discovery of isokurarinone as an ATCase-engaging lead with potent activity against methicillin-resistant *Staphylococcus aureus*

**DOI:** 10.1080/21505594.2026.2707766

**Published:** 2026-07-21

**Authors:** Xinyuan Cao, Xiaorong Yang, Lixia Dai, Xueyan Liu, Zile Gong, Xiaoyan Yu, Yuchao Ma, Tong Bu, Haowen Wu, Xiaolou Miao, Xiaofei Shang, Changcai Bai

**Affiliations:** a School of Pharmacy, Ningxia Medical University, Yinchuan, China; b Key Laboratory of Veterinary Pharmaceutical Development of Ministry of Agriculture, Key Laboratory of New Animal Drug Project, Lanzhou Institute of Husbandry and Pharmaceutical Sciences, Chinese Academy of Agricultural Sciences, Lanzhou, Gansu Province, China

**Keywords:** Methicillin-resistant *Staphylococcus aureus*, isokurarinone, aspartate transcarbamoylase, pyrimidine biosynthesis, natural product

## Abstract

The global proliferation of methicillin-resistant *Staphylococcus aureus* (MRSA) persists as a significant contributor to challenging infections, highlighting the urgent necessity for therapies that utilize novel mechanisms. Aspartate transcarbamoylase (ATCase), which catalyzes the initial committed step of *de novo* pyrimidine biosynthesis, represents a promising metabolic target with potential relevance to MRSA fitness and persistence. Through structure-based virtual screening and experimental validation, we identified the flavonoid isokurarinone as a compound targeting ATCase, demonstrating potent anti-MRSA activity. Docking and molecular dynamics simulations, along with surface plasmon resonance and differential scanning fluorimetry, provided evidence for direct binding, which was accompanied by a decrease in *pyrB* expression and a reduction in ATCase activity. Non-targeted metabolomics revealed a disruption of pyrimidine nucleotide homeostasis, coinciding with impaired membrane integrity, reduced proton motive force, decreased intracellular ATP levels, and increased oxidative stress. Isokurarinone also showed an additive interaction with vancomycin *in vitro*, inhibited biofilm formation and altered the expression of virulence-associated genes. Safety evaluation showed cell-type-dependent cytotoxicity in mammalian cells, while no obvious acute oral toxicity was observed in mice at a single dose of 2 g/kg. In a murine model of MRSA-infected wounds, isokurarinone accelerated wound closure, reduced bacterial burden, and attenuated local inflammatory mediators. Collectively, these findings support the notion of ATCase as a metabolism-guided target for MRSA and nominate isokurarinone as a promising lead scaffold for therapeutic development.

## Introduction

The clinical persistence of methicillin-resistant *Staphylococcus aureus* (MRSA) reflects a synergistic interplay between antimicrobial resistance and virulence programs that promote immune evasion, tissue persistence, and chronic infection. Central to this resilience is biofilm formation, which creates a protected niche that enhances tolerance to antimicrobial exposure and host defenses, contributing to recurrent and device-associated infections [1,2]. These characteristics underscore the limitations of conventional bactericidal therapies and motivate the development of pathogen-disarming strategies that attenuate infection-relevant functions while potentially reducing the selective pressure for resistance evolution [3]. Concurrently, targeting metabolic nodes may compromise both bacterial fitness and persistence-associated virulence traits, offering a complementary approach to treating chronic MRSA infections.

A promising strategy to diminish virulence-associated persistence is to target metabolic pathways that sustain infection-relevant physiology. The *de novo* pyrimidine biosynthesis pathway provides essential precursors for nucleic acid synthesis and cell envelope biogenesis [4,5]. Increasing evidence links pyrimidine availability to virulence-associated phenotypes, including regulatory circuits and biofilm development [6,7]. Aspartate transcarbamoylase (ATCase, encoded by *pyrB*), the enzyme catalyzing the first committed step of this pathway, serves as a critical node controlling pyrimidine flux. Perturbation of *de novo* pyrimidine synthesis has been suggested to reprogram virulence regulation, influencing structural determinants such as biofilm formation and potentially toxin-linked programs [8]. Consistent with this concept, pyrimidine nucleotide homeostasis has been mechanistically linked to robust MRSA biofilm development [9]. Furthermore, pharmacological interference with *de novo* pyrimidine synthesis suppresses MRSA biofilm formation and virulence-associated outputs, while functional rescue through exogenous pyrimidine supplementation supports a causal role of nucleotide homeostasis [6]. Therefore, targeting pyrimidine biosynthesis, particularly ATCase, may provide a viable strategy to weaken the metabolic capacity that supports both biofilm-associated persistence and toxin-linked virulence [10].

Natural products provide privileged chemical scaffolds for probing vulnerabilities and can reveal tractable antibacterial targets. Flavonoids exhibit broad bioactivities and have demonstrated efficacy against drug-resistant pathogens [11]. Notably, multiple flavonoids interfere with biofilm formation across various species [12,13]. However, the extent to which a natural product scaffold can engage MRSA ATCase and translate target engagement into coordinated modulation of virulence circuitry and downstream effectors remains insufficiently defined.

In this study, we employed target-guided virtual screening to prioritize flavonoids predicted to bind MRSA ATCase, identifying isokurarinone for experimental evaluation. By integrating computational and experimental approaches, we established isokurarinone as a stable ATCase-binding agent with strong antibacterial efficacy against MRSA both *in vitro* and *in vivo*, and further investigated its potential mechanism of action. In addition, biofilm formation, mature biofilm disruption, and toxin- or virulence-associated gene expression were examined to determine whether isokurarinone also affects related phenotypes. To further assess its translational potential, we evaluated its *in vitro* interaction with vancomycin and performed preliminary safety assessments, including mammalian cell cytotoxicity and acute oral toxicity in mice. The therapeutic relevance of isokurarinone was subsequently investigated in a murine model of MRSA-infected wounds. Collectively, these findings support ATCase as a tractable antibacterial target and nominate isokurarinone as a mechanism-guided lead scaffold for developing new interventions against biofilm-associated MRSA infections.

## Method

### Chemicals

Licochalcone D, isokurarinone, sophoranone G, kurarinone and methylophiopogonanone A were purchased from TargetMol (USA) with at least 98% purity. Norfloxacin was purchased from Shandong Sparkjade Scientific Instruments Co., Ltd (China). Vancomycin was purchased from Shanghai Macklin Biochemical Co., Ltd (China). Stocks of all antibiotics of 6.4 mg/mL were made in DMSO for subsequent experiments.

### Bacterial strains and growth conditions

*Staphylococcus aureus* ATCC43300 (MRSA), *Staphylococcus aureus* ATCC29213, *Enterococcus faecalis* ATCC29212, *Escherichia coli* ATCC25922, *Proteus vulgaris* ATCC49132, *Pseudomonas aeruginosa* ATCC27318, and *Salmonella typhimurium* ATCC14028 were sourced from a frozen stock maintained at the Lanzhou Institute of Husbandry and Pharmaceutical Sciences (Table S1), which were purchased from ATCC (USA). The study tested 45 clinical MRSA isolates obtained from dairy farms across four provinces in China [14]. All strains were cultured aerobically at 37°C in cation-adjusted Mueller-Hinton broth (CAMHB), Brain-Heart Infusion broth (BHI), Tryptic soy broth (TSB) and Lysogeny broth (LB).

### Virtual screening

Virtual screening was performed using AutoDock Vina on the Yinfo Cloud Computing Platform. The ATCase structure (PDB ID: 6PNZ) was prepared in AutoDockTools by adding hydrogens and charges. Compounds from the L6120 Flavonoid Natural Product Library (517 compounds) were docked against ATCase. The docking site was defined around the catalytic cavity corresponding to the co-crystallized ligand region, with the grid center at (6.102, 52.447, 63.065) Å and a box size of (51.173, 45.389, 45.897) Å. AutoDock Vina was used for primary docking, and top-ranked candidates were re-docked/rescored using DOCK 6.9 as an orthogonal validation step.

### Molecular docking analysis

Docking poses were visualized and analyzed using Mol* Viewer. Candidate poses were inspected to confirm occupancy of the catalytic cavity and to map putative hydrogen-bonding and hydrophobic contacts with key residues. Ligand binding poses were generated with AutoDock Vina, and the top-ranked conformations were inspected to identify plausible interaction patterns within the ATCase pocket using Mol* Viewer [15].

### RNA extraction and quantitative reverse transcription PCR (RT-qPCR) analysis

Logarithmic-phase MRSA cultures were treated with isokurarinone at 1/2 × MIC and harvested at 8 h (regulatory/toxin-associated genes) or 24 h (biofilm-associated genes), as specified in the figure legends. Total RNA was extracted and reverse-transcribed into cDNA for RT-qPCR. Relative expression was quantified using the 2^-ΔΔCt method with 16S rRNA as the internal reference gene, and results are reported as fold change vs the vehicle-treated control (Primers were listed in Table S2).

### ATCase activity assay in MRSA lysates

Logarithmic-phase MRSA cultures were treated with isokurarinone at 1/2 × MIC, 1 × MIC and 4 × MIC for 8 h and lysed in PBS containing lysozyme. Lysates were clarified by centrifugation and normalized by total protein (BCA). ATCase activity was then quantified using a commercial ATCase activity assay kit (Yuanju BioTech Center, Shanghai) according to the manufacturer’s instructions.

### Molecular dynamics simulation

Molecular dynamics (MD) simulations were carried out in GROMACS 2024 using docking-derived ATCase-isokurarinone complexes as starting conformations. The protein was parameterized with the Amber14SB force field, whereas ligand parameters were assigned using GAFF. The solvated complexes were equilibrated sequentially under NVT and then NPT conditions at 300 K for 200 ps per step. Subsequently, a 100 ns production MD run was performed to sample the conformational dynamics of the complex and to characterize ATCase-isokurarinone interaction stability over time. The MD workflow followed standard protocols as described previously [16].

### Differential scanning fluorometry experiments

The intrinsic tryptophan fluorescence of the protein was monitored in real time at 330 nm and 350 nm using a Prometheus NT.48 instrument with an excitation wavelength of 285 nm. 1 μM solution of ATCase in PBS was loaded into capillaries (10 μL per capillary) and subjected to a temperature ramp from 20°C to 95°C at a rate of 5°C/min. Fluorescence emission at 330 nm and 350 nm was recorded throughout the heating process, and the protein melting temperature was determined accordingly [17].

### Surface plasmon resonance

In brief, ATCase protein was immobilized in CM5 sensor chip, blocked with ethanolamine, and then equilibrated with PBS. Isokurarinone stock solution (10 mM) was diluted to a series of concentrations (10, 5, 2.5, 1.25, 0.625 and 0.3125 μM). At the end of each flow, cells were regenerated for 5 min with glycine-HCl solution. Association and dissociation constants were obtained by global fitting of the data to a 1:1 Langmuir binding model using BIAcore Insight Evaluation software (Cytiva, V6.0 Marlborough, MA, USA). Data were exported to Prism10 software for generating the final figures [18].

### Minimum inhibitory concentration (MIC) assay

The MIC of potential lead candidates was determined by broth microdilution. Candidates were initially diluted to 64 μg/mL, and 200 μL was added to the first column of a 96-well plate containing 100 μL CAMHB in columns 2–12. Two-fold serial dilutions were performed across the plate, generating a final concentration range of 64 to 0.0625 μg/mL. Then, 100 µL MRSA cultures was added, which had been adjusted to a 0.5 McFarland standard and subsequently subjected to a 1:100 dilution. Each concentration was tested in triplicate against the target strain. Following an 18-hour incubation period at 37°C, the MIC was defined and recorded as the lowest concentration at which no visible bacterial growth was observed [19].

### Growth curve assay

A 1:1000 dilution of overnight MRSA cultures was mixed with isokurarinone (1/2 × MIC, 1 × MIC, 2 × MIC, 4 × MIC) and further cultured in a 37°C incubator for 24 h. During incubation, the absorbance (OD) value at 600 nm was measured with a microplate reader at various time points to obtain the bacterial growth curve.

### Time-kill assay

MRSA cultures were adjusted to 0.5 McFarland, diluted 1:100, then incubated in TSB containing isokurarinone at concentrations of 1/2 ×, 1 ×, 2 ×, and 4 × MIC (37°C, 150 rpm). Aliquots (200 μL) were collected at 0, 1, 2, 4, 6, 8, 10, 12, and 24 h, serially diluted in TSB, and plated onto MHA for CFU determination following incubation at 37°C for 18 h. All assays were conducted in triplicate [19].

### Resistance development

To assess the potential for resistance development, MRSA cultures capable of growth at the highest isokurarinone concentration were diluted 1:10000 and passaged daily into fresh 96-well plates containing CAMHB with serially diluted isokurarinone. This serial passage was continued for 20 days, with Norfloxacin and Vancomycin serving as a control antibiotic. Changes in MIC were monitored throughout the passage period [19].

### Biofilm formation assay

MRSA cultures were exposed to TSB supplemented with isokurarinone at concentrations corresponding to 1/2 ×, 1 ×, 2 ×, and 4 × MIC for 18 h at 37°C in 96-well plates, after which planktonic cells were removed, adherent biofilms washed with PBS, stained with 0.1 % crystal violet for 30 min, rinsed, and subsequently solubilized with 95 % ethanol before absorbance measurement at 595 nm for quantification relative to untreated controls [20].

### Biofilm eradication assay

After 24 h biofilm maturation, supernatants were removed and replaced with fresh TSB containing isokurarinone (1/2 ×, 1 ×, 2 ×, and 4 × MIC), followed by incubation for an additional 24 h. Residual biofilm biomass was quantified by crystal violet staining and absorbance at 595 nm, reported as a percentage of the untreated control [21].

### Hemolysis test

Fresh sheep red blood cells (RBCs) were washed three times with sterile PBS (1200 rpm, centrifuged at 4°C for 5 min) and then added to 96-well plates containing isokurarinone, making a concentration of 2 % for RBCs and 1/2 × MIC, 1 × MIC, 2 × MIC, 4 × MIC isokurarinone, with a final volume of 200 μL. PBS treated RBCs were used as negative control, and 0.5 % Triton X100 treated RBCs were used as positive control. After incubation at 37°C for 3 h, the mixtures were centrifuged at 1200 g for 15 min at 4°C. The supernatants were collected in new 96-well plates with a flat bottom, and OD_540_ was measured. All experiments were performed with three independent replicates. The rate of hemolysis was calculated according to the following equation [13].

(1) $$\begin{array}{rl} & \mathrm{Hemolysis}(\%)=\\ & \;\frac{\mathrm{OD}540(\mathrm{Isokurarinone})-\mathrm{OD}540(\mathrm{Negative})}{\mathrm{OD}540(\mathrm{Positive})-\mathrm{OD}540(\mathrm{Negative})}\times 100\% \end{array}$$

### Transmission electron microscopy (TEM)

Logarithmic-phase MRSA cultures were incubated with 4 × MIC isokurarinone at 37°C for 12 h and washed three times with PBS. Samples were pre-fixed overnight at 4°C with 3 % glutaraldehyde in PBS, washed twice with PBS, and post-fixed with 1 % osmium tetroxide for 3 h at room temperature. After three additional PBS washes, specimens were dehydrated through a graded acetone series, infiltrated with acetone-Epon812 mixtures (3:1, 1:1, 1:3), and embedded in pure Epon812 at 60°C for 48 h. Ultrathin sections (60–90 nm) were collected on copper grids, stained with uranyl acetate and lead citrate, and imaged using a TEM (JEM-1400FLASH, JEOL, Japan).

### Scanning electron microscope (SEM)

Logarithmic-phase MRSA cultures were incubated with 4 × MIC isokurarinone at 37°C for 12 h and washed three times with PBS. Then prefixed with 2 mL of 3 % glutaraldehyde (v/v) in PBS at 4°C overnight. Gradient dehydration was performed using ethanol solutions of varying concentrations. They were then replaced with isoamyl acetate, and the bacterial cells were dried overnight with liquid CO_2_, sprayed with thin-layer gold, and visualized by SEM (JSM-IT700HR, JEOL, Japan) [22].

### Cell membrane integrity analysis

To evaluate membrane integrity following exposure to isokurarinone at 1/2 ×, 1 ×, 2 ×, and 4 × MIC concentrations for 2 h at 37°C with shaking at 180 rpm, MRSA cultures were harvested by centrifugation, washed with PBS, and subsequently stained with either propidium iodide (PI, 10 μg/mL) or DAPI (1 μg/mL) for 30 min at 37°C before fluorescence intensity measurement using an EnSpire 2300 Multimode Plate Reader (Perkin Elmer, Rodgau, Germany) [23].

### Bacterial membrane permeability

Logarithmic-phase MRSA cultures were treated with isokurarinone for 1 h at 37°C and centrifuged (5000 rpm, 10 min). The supernatant protein concentration was quantified using a BCA protein assay in a 96-well plate format. The co-culture supernatant was collected from each group and mixed with 2-nitrophenyl-β-D-galactopyranoside (3 mM), followed by additional incubation at 37°C for 2 h. The extracellular β-galactosidase activity was determined by measuring the absorbance at 420 nm [24,25]. The absorbance was measured using a Multiskan Go Microplate Spectrophotometer (Thermo Scientific., USA).

### Proton motive force assay

The proton motive force of logarithmic-phase MRSA cultures treated by isokurarinone was measured with pH-sensitive fluorescence probe BCECF-AM (20 × 10^−6^ m). After the fluorescence stabilized, 1/2 ×, 1 ×, 2 ×, and 4 × MIC isokurarinon were added. For all BCECF experiments, the excitation and emission wavelengths on the fluorescence spectrometer were set to 500 and 522 nm, using a EnSpire 2300 Multimode Plate Reader (Perkin Elmer, Rodgau, Germany) [26,27].

### Determination of ATP

Isokurarinone was added to the logarithmic-phase MRSA cultures at the final concentration of 1/2 ×, 1 ×, 2 ×, and 4 × MIC, respectively and then cultured for 2 h (37°C, 180 rpm). The culture medium was centrifuged at 5000 g for 5 min and rinsed with sterile PBS. MRSA precipitates were lysed by lysozyme, centrifuged and the supernatant was prepared for intracellular ATP levels measurement. Detecting solution was added to a 96-well plate and incubated at room temperature for 5 min. Subsequently, the supernatants were added to the well, mixed quickly, and the luminescence was measured by EnSpire 2300 Multimode Plate Reader (Perkin Elmer, Rodgau, Germany) [26,28].

### Intracellular ROS detection

Logarithmic-phase MRSA cultures loaded with DCFH-DA for 30 min in the dark at 37°C. Isokurarinone was added to final concentrations ranging from 1/2 × to 4 × MIC and incubated for 2 h at 37°C with shaking at 180 rpm. Bacteria were pelleted, washed with PBS, and fluorescence was quantified using an EnSpire 2300 Multimode Plate Reader (Perkin Elmer, Rodgau, Germany) [28].

### Nucleic acid and protein leakage

The mid-logarithmic phase of bacteria was suspended in PBS at a bacterial density of 10 × 10^8^ CFU/mL, and incubated with different concentrations of isokurarinone solutions at 37°C. At different time points, aliquots of co-incubation were taken and centrifuged to measure the absorbance of supernatant at 260 nm and 280 nm using a microplate reader [29].

### Metabolomic analysis

Logarithmic-phase MRSA cultures were treated in parallel with either 1/2 × MIC isokurarinone or an equal volume of solvent (Control). The samples were incubated for 4 h at 37°C and 180 rpm in a shaking incubator. After centrifugation, the culture medium was discarded and the bacterial pellet was washed thrice with 10 mL of pre-chilled sterile PBS buffer. The bacterial metabolism was quenched using liquid nitrogen. Metabolite profiling and metabolomic data analyses were performed at Shanghai MajorbioBio pharm Biotechnology Co., Ltd. (Shanghai, China) [28].

### Cytotoxicity assay

RAW264.7 cells were seeded into 96-well plates at 5 × 10^3^ cells per well in 100 μL medium and allowed to adhere for 24 h. Cells were then treated with various concentrations of isokurarinone for 24 h. Subsequently, 10 μL of CCK-8 reagent was added to each well and incubated for 1 h at 37°C. Cell viability was assessed by measuring the absorbance at 450 nm with a microplate reader [14].

### Acute oral toxicity assessment

A preliminary acute oral toxicity assessment was performed using a limit dose. Twelve BALB/c mice were randomly assigned to a vehicle control group and an isokurarinone-treated group, with six mice per group, including three males and three females. Isokurarinone was administered once by oral gavage at 2 g/kg with a gavage volume of 0.2 mL/20 g body weight. The vehicle control group received an equal volume of the same vehicle. After administration, mice were closely observed during the first 24 h and then monitored daily for 14 days. Mortality, clinical signs, behavioral abnormalities, food and water intake, and body weight changes were recorded. At the end of the observation period, blood samples and major organs were collected for organ coefficient, biochemical, and histopathological analyses [30].

### Combination with antibiotics

The interaction between isokurarinone and vancomycin was evaluated using a checkerboard microdilution assay. Two-fold serial dilutions of isokurarinone and vancomycin were prepared in MH broth in 96-well plates. Isokurarinone was diluted along one axis, and vancomycin was diluted along the other axis. The final concentrations ranged from 0.125 to 8 μg/mL for isokurarinone and from 0.0625 to 4 μg/mL for vancomycin. MRSA suspension was added to each well to obtain a final inoculum of approximately 5 × 10^5^ CFU/mL. After incubation at 37°C for 18 h, bacterial growth was assessed visually. The fractional inhibitory concentration index (FICI) was calculated as follows: FICI = MIC of isokurarinone in combination/MIC of isokurarinone alone + MIC of vancomycin in combination/MIC of vancomycin alone. FICI ≤ 0.5 was interpreted as synergy, 0.5 < FICI ≤1.0 as an additive effect, 1.0 < FICI ≤4.0 as an indifferent effect, and FICI > 4.0 as antagonism. All experiments were performed with three independent biological replicates [31].

### Mouse wound healing assay

Sixty male BALB/c mice (6 to 8 weeks, 18 to 22 g) were purchased from Lanzhou Veterinary Research Institute, Chinese Academy of Agricultural Sciences, which were randomly allocated to five groups (*n* = 12 per group): Control (uninfected, PBS), Model (MRSA-infected, PBS), Iso-H (MRSA-infected, isokurarinone, 20 mg/mL), Iso-L (MRSA-infected, isokurarinone, 5 mg/mL), and Mup (MRSA-infected, mupirocin, 20 mg/g). Mice were anesthetized with isoflurane and adequate anesthesia was confirmed by loss of the pedal withdrawal reflex. After dorsal shaving, a 10-mm full-thickness excisional wound was created and inoculated with 20 μL MRSA (1 × 10^8^ CFU per wound), then covered with Tegaderm. At 48 h post-infection, topical treatment was initiated: isokurarinone in 1 % DMSO/PEG400 (Model received the same vehicle) or mupirocin in PEG400/PEG3350. A 20 μL dose was applied per wound (0.4 mg Iso-H; 0.1 mg Iso-L) twice daily for 7 days and once daily for the next 4 days. Wounds were photographed on days 0, 3, 7, and 11 and closure quantified using ImageJ. On day 7, wound tissues were collected for CFU enumeration and cytokine ELISA; on day 11, tissues were collected for gene expression analysis. Wound tissues harvested on days 7 and 11 were fixed in 4 % paraformaldehyde for H&E and Masson staining. Local tolerability (erythema/edema/necrosis) and body weight were monitored throughout [32,33].

### Statistical analysis

Data are presented as mean ± SD from at least three independent biological replicates unless otherwise stated. Statistical analyses were performed using one-way or two-way ANOVA with GraphPad Prism 10.1.2 software. Statistical significance was defined by *p < 0.05, **p < 0.01, ***p < 0.001. No animals were excluded from analysis based on experimental outcomes.

## Results

### Identification of isokurarinone as an ATCase-binding candidate against MRSA

**Figure 1.**
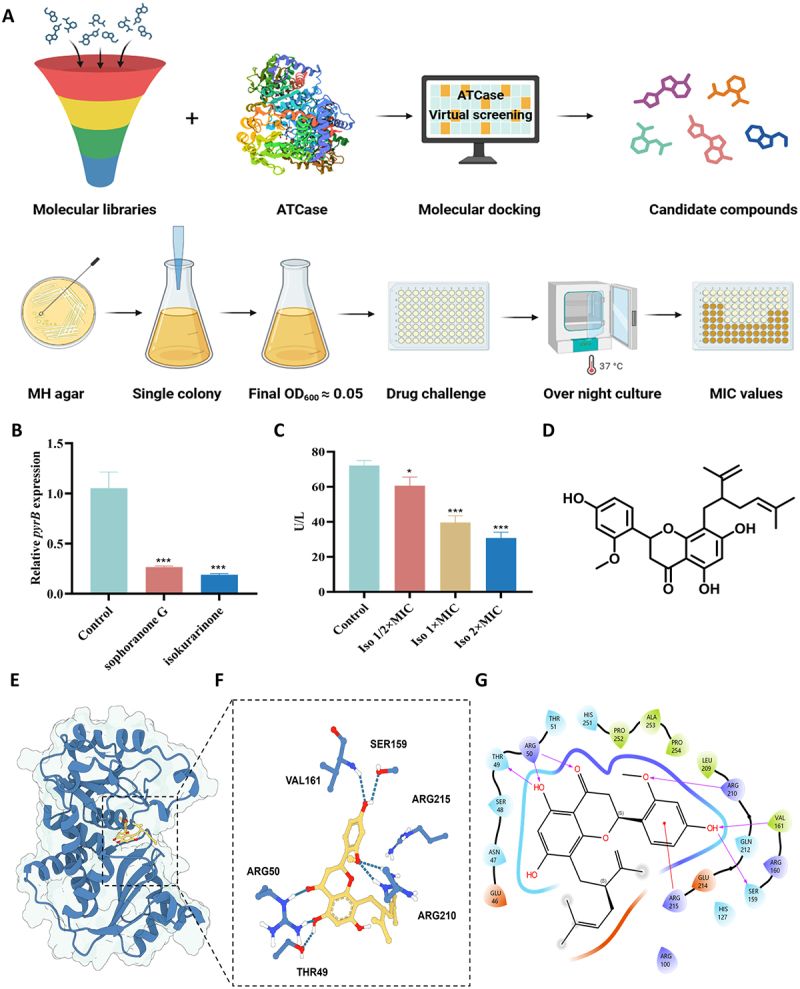
Structure-guided identification of isokurarinone as an ATCase-targeting hit in MRSA. (A) Workflow of structure-based virtual screening. (B) Relative pyrB expression after isokurarinone treatment. (C) ATCase activity in MRSA lysates. (D) Chemical structure of isokurarinone. (E) Predicted binding mode of isokurarinone with ATCase. (F) Magnified binding pocket view. (G) Two-dimensional interaction map. Data are shown as mean ± SD (*n* = 3). Statistical analysis was performed using one-way ANOVA (*p < 0.05, ***p < 0.001).

The flavonoid natural product library was selected for focused screening because flavonoids are structurally diverse, experimentally accessible, and widely reported to possess antibacterial activity. In particular, prenylated flavonoids have attracted attention as anti-MRSA scaffolds due to their enhanced lipophilicity and bioactivity. Although a broader compound database was initially considered for virtual screening, many top-ranked hits were limited by availability and feasibility for experimental validation. Therefore, we focused on L6120 Flavonoid Natural Product molecular libraries to identify accessible ATCase-binding candidates with antibacterial potential (Figure 1(A)). The compounds licochalcone D, isokurarinone, kurarinone, sophoranone G, and methylophiopogonanone A exhibited the highest Grid Scores of −66.61, −62.48, −57.60, −55.55, and −55.23 kcal/mol, respectively (Table 1). To assess the accuracy of the prediction results, we evaluated the minimum inhibitory concentrations (MICs) of the five selected candidates against pathogenic bacteria. As illustrated in Table 2, methylophiopogonanone A demonstrated no antibacterial activity against either Gram-positive or Gram-negative strains, with an MIC exceeding 64 µg/mL. In contrast, isokurarinone, licochalcone D, kurarinone, and sophoranone G exhibited antibacterial effects exclusively against Gram-positive bacteria, with no activity detected against Gram-negative strains. Among these, isokurarinone and sophoranone G displayed the most potent activity against MRSA, with an MIC of 4 μg/mL. In comparison, licochalcone D and kurarinone showed only moderate antibacterial efficacy, with MICs of 32 μg/mL and 16 μg/mL, respectively.

**Table 1.** The 5 top-ranked candidates screened from the L6120 flavonoid natural product library database.

Number	CAS	Name	Grid_Score	Grid_vdw	Grid_es	Int_energy
1	144,506-15–0	licochalcone D	−66.61	−54.37	−12.24	7.76
2	52,483–02-0	isokurarinone	−62.48	−50.20	−12.28	22.69
3	97,938–30-2	sophoranone G	−57.60	−44.50	−13.10	30.88
4	34,981–26-5	kurarinone	−55.55	−45.91	−9.64	20.36
5	74,805–92-8	methylophiopogonanone A	−55.23	−50.32	−4.91	9.75

**Table 2.** MIC of 5 top-ranked candidates against pathogenic bacteria (μg/mL).

Strains	licochalcone D	isokurarinone	sophoranone G	kurarinone	methylophiopogonanone A
*aureus* ATCC43300	32	4	4	16	>64
*S. aureus* ATCC29213	32	4	4	16	>64
*E. faecalis* ATCC29212	32	2	4	16	>64
*E. coli* ATCC25922	>64	>64	>64	>64	>64
*P. vulgaris* ATCC49132	>64	>64	>64	>64	>64
*P. aeruginosa* ATCC27318	>64	>64	>64	>64	>64
*S. typhimurium* ATCC14028	>64	>64	>64	>64	>64

The *pyrB* gene encodes aspartate transcarbamoylase (ATCase), which catalyzes the initial committed step in bacterial pyrimidine biosynthesis. This pathway is responsible for the production of essential nucleotides, including cytosine, thymine, and uracil, which serve as the fundamental building blocks of DNA and RNA [34]. Consequently, we employed RT-qPCR to assess the effects of sophoranone G and isokurarinone on *pyrB* expression. As illustrated in Figure 1(B), isokurarinone at 1/2×MIC resulted in a more pronounced reduction in *pyrB* expression compared to sophoranone G. Furthermore, the activity of ATCase was quantified using ELISA. As depicted in Figure 1(C), isokurarinone at concentrations of 1/2 ×, 1 ×, and 2 × MIC (Figure 1(D)) significantly inhibited ATCase activity. It has been reported that isokurarinone, a prenylated flavonoid primarily derived from *Sophora flavescens*, exhibits inhibitory effects against *Staphylococcus aureus* and *Bacillus subtilis* [35]. Based on the aforementioned studies and existing literature, isokurarinone was selected as the final candidate compound for further investigation due to its favorable binding stability and specificity with ATCase, as well as its excellent *in vitro* anti-MRSA activity and inhibitory effects on both the expression of its encoding gene and enzyme activity.

Molecular docking was employed to investigate the binding modes of isokurarinone to ATCase. As illustrated in Figure 1(E), isokurarinone interacts within the active cavity of ATCase through multiple non-covalent interactions. Its core skeleton establishes several hydrogen bonds with THR49 and ARG50, while the m-dihydroxyphenyl group in the side chain forms additional hydrogen bonds with SER159, VAL161, and ARG210 (Figure 1(F)). Concurrently, hydrophobic amino acids within the binding pocket, such as PRO254, enhance the binding affinity of isokurarinone to ATCase through hydrophobic interactions. As shown in Figure 1(G), two-dimensional interaction analyses further indicate a potential cation-π interaction between isokurarinone and the side chain of ARG215. The synergistic effect of these interactions forms the structural basis for the exceptional binding capacity of isokurarinone. Overall, this binding mode demonstrates favorable stability and specificity. However, the dynamic stability of the bound conformation necessitates validation through further molecular dynamics simulations.

### Molecular dynamics analysis confirms the stable binding of isokurarinone to ATCase

**Figure 2.**
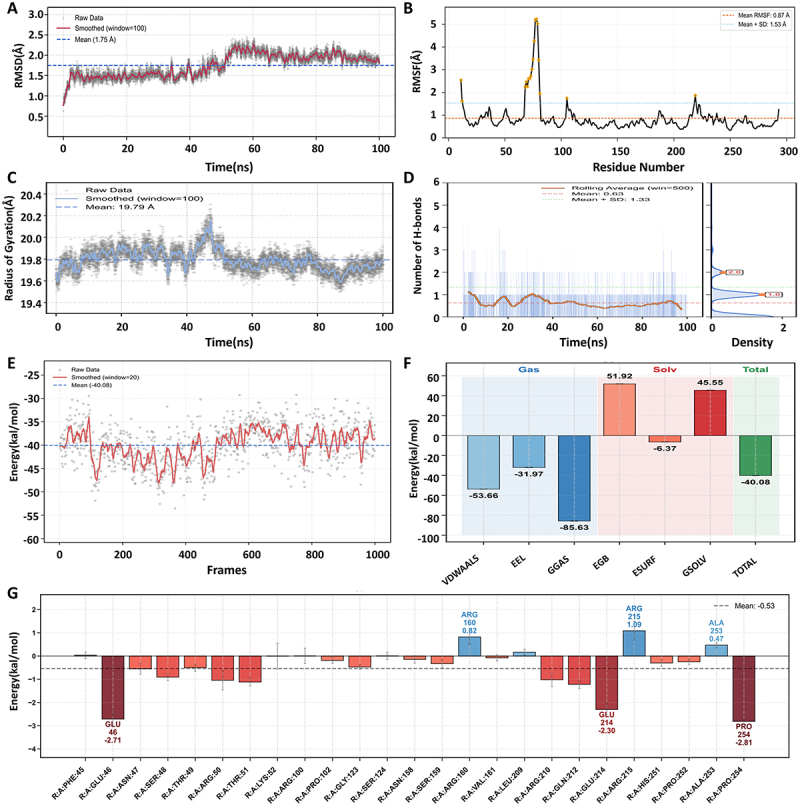
Molecular dynamics simulation and binding energy analysis of the ATCase-isokurarinone complex. (A) RMSD curves. (B) Residue RMSF values. (C) Rg of backbone atoms. (D) Hydrogen bond numbers between isokurarinone and ATCase. (E) Total binding energy of the ATCase-isokurarinone complex. (F) MM-GBSA free energy decomposition. (G) Energy contributions of key binding-site residues.

Molecular dynamics simulation plays a pivotal role in diverse scientific fields, particularly in investigating biomolecular interactions and the physical dynamics of macromolecules [36]. In this study, we systematically investigate the interaction mode between isokurarinone and ATCase, along with its stability and energetics during molecular dynamics simulations [37]. RMSD analysis revealed that the isokurarinone-ATCase complex equilibrated into a stable state, characterized by a bimodal distribution averaging 1.75 Å (Figure 2(A)). RMSF analysis confirmed the high stability of the core binding region, with most residues exhibiting limited fluctuations (<1.50 Å) (Figure 2(B)). RG analysis consistently indicated a stable, compact conformation for the protein-ligand complex throughout the simulation (Figure 2(C)). Despite a low average hydrogen bond count (0.63), the binding was stabilized by persistent interactions with key residues, including ARG210, ARG100, and ASN47 (Figure 2(D)). Ramachandran plot analysis revealed that the secondary structure of ATCase maintains high conservation throughout the simulation process, with stable proportions of α-helices and β-sheets. No significant rearrangement of secondary structures occurred, further supporting the overall stability of the system (Figure S1). In terms of energy dynamics, PCA and FEL analyses showed that the system exhibits two energy valley minima during the simulation, corresponding to two metastable structures (Figure S2).

MM/GBSA calculations yielded a total binding free energy of −40.08 kcal/mol (Figure 2(E)), which is primarily driven by van der Waals interactions (−53.66 kcal/mol) and electrostatic interactions (−31.97 kcal/mol). In contrast, polar solvation energy (+51.92 kcal/mol) serves as an unfavorable factor for binding (Figure 2(F)). Residue decomposition analysis further indicates that residues such as GLU46, GLU214, and PRO254 make significant contributions during the binding process, potentially representing key sites for subsequent drug optimization (Figure 2(G)).

### Comprehensive evaluation of isokurarinone-ATCase binding

**Figure 3.**
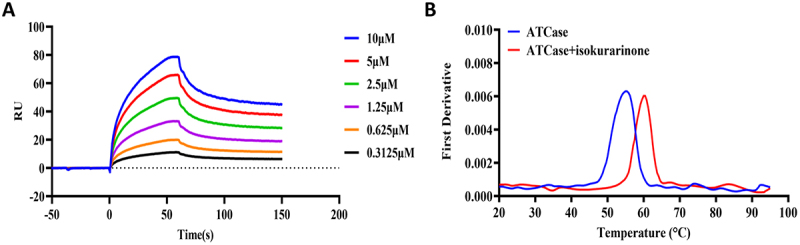
Comprehensive evaluation of isokurarinone-ATCase binding. (A) SPR analysis of direct isokurarinone-ATCase binding. (B) DSF analysis of ATCase thermal stability with or without isokurarinone.

We utilized the ATCase protein previously expressed and purified by our research group to investigate the direct interaction between isokurarinone and ATCase. ATCase was immobilized on a BIAcore CM5 sensor chip, and its interaction with isokurarinone was analyzed using a surface plasmon resonance (SPR) assay, as depicted in Figure 3(A). The SPR measurements yielded an estimated dissociation constant (K_D_) of 2.45 μM, with an association rate constant (K_a_) of 1.69 M^−1^ s^−1^, confirming a dose-dependent direct interaction between isokurarinone and ATCase. To further validate the binding, we observed the intracellular binding of isokurarinone to the ATCase protein using differential scanning fluorimetry (DSF). As shown in Figure 3(B), the melting temperature (T_m_) of ATCase increased from 55°C to 60°C in the presence of 10 μM isokurarinone, indicating enhanced thermal stability. In summary, these results suggest that isokurarinone can bind to ATCase.

### Antibacterial property of isokurarinone in vitro

**Figure 4.**
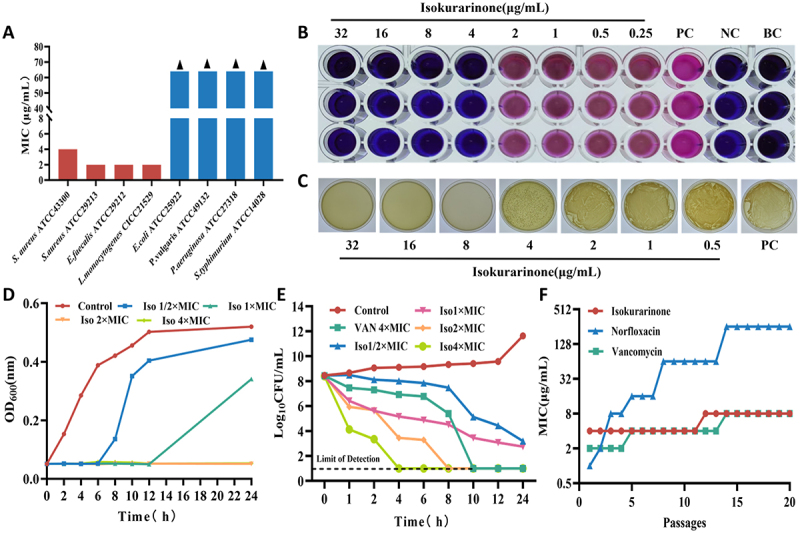
*In vitro* antibacterial activity of isokurarinone. (A) Antibacterial activity against different pathogenic bacteria. (B) MIC against MRSA. PC, positive control; NC, negative control; BC, blank control. (C) MBC against MRSA. (D) Growth curve. (E) Time-kill curve. (F) Resistance development assay. Data are presented as mean ± SD (*n* = 3).

Subsequently, we determined the minimum inhibitory concentration (MIC) of isokurarinone against various bacterial strains, including both Gram-negative and Gram-positive bacteria. The results indicated that the MICs of isokurarinone for Gram-negative bacteria, such as *P. vulgaris*, *E. coli*, *S. typhimurium*, and *P. aeruginosa*, exceeded 64 μg/mL. In contrast, isokurarinone exhibited the most potent antibacterial activity against Gram-positive bacteria, with MIC values ranging from 2 to 4 μg/mL (Figure 4(A)). Notably, the MIC of isokurarinone against methicillin-resistant *Staphylococcus aureus* (MRSA) was 4 μg/mL, while the minimum bactericidal concentration (MBC) was 8 μg/mL (Figure 4(B,C)). Besides the standard strains, isokurarinone also demonstrated significant antibacterial activity against clinically isolated strains, with MICs ranging from 2 to 4 µg/mL (Table 3). Growth curve analysis revealed a concentration-dependent effect at 1/2 × MIC and 1 × MIC, isokurarinone prolonged the logarithmic phase, delaying the transition to the stationary phase. At higher concentrations (2 × MIC and 4 × MIC), it completely inhibited bacterial growth (Figure 4(D)). To further assess the antimicrobial potency of isokurarinone, a killing kinetic assay was conducted, revealing that isokurarinone at 4 × MIC eradicated all MRSA cells within 6 h (Figure 4(E)).

**Table 3.** Antibacterial activity against clinical isolates of MRSA (μg/mL).

Strains	isokurarinone	vancomycin	ampicillin
1#	4	4	>64
2#	2	2	>64
3#	2	4	>64
4#	2	2	>64
5#	2	2	>64
6#	4	4	>64
7#	2	4	>64
8#	2	4	>64
9#	1	4	>64
10#	2	4	>64
11#	2	2	>64
12#	2	4	>64
13#	2	2	>64
14#	2	4	>64
15#	4	4	>64
16#	4	4	>64
17#	2	2	>64
18#	2	4	>64
19#	4	8	>64
20#	2	8	>64
21#	2	4	>64
22#	2	4	>64
23#	2	4	>64
24#	4	4	>64
25#	2	4	>64
26#	4	4	>64
27#	2	4	>64
28#	2	4	>64
29#	2	4	>64
30#	2	4	>64
31#	2	4	>64
32#	2	4	>64
33#	2	2	>64
34#	4	4	>64
35#	4	4	>64
36#	4	4	>64
37#	4	4	>64
38#	4	4	>64
39#	4	8	>64
40#	4	8	>64
41#	4	8	>64
42#	4	8	>64
43#	4	8	>64
44#	4	8	>64
45#	4	8	>64

Subsequently, MRSA was continuously exposed to isokurarinone at 1/2 × MIC for 20 days to evaluate resistance development. As shown in Figure 4(F), the MIC of isokurarinone increased only 2-fold during serial passage, whereas the MICs of Norfloxacin and Vancomycin increased 256-fold and 4-fold, respectively. These results indicate that isokurarinone exhibited a relatively low tendency to induce resistance development under the tested conditions. To further evaluate the potential compatibility of isokurarinone with conventional anti-MRSA antibiotics, a checkerboard assay was performed using vancomycin. The combination of isokurarinone and vancomycin showed an additive interaction against MRSA, with 0.5 < FICI ≤1.0, indicating no antagonistic effect under the tested conditions (Figure S3). Collectively, these findings establish that isokurarinone possesses the capability to act as a highly effective anti-MRSA agent.

### The potential mechanism of isokurarinone against MRSA

**Figure 5.**
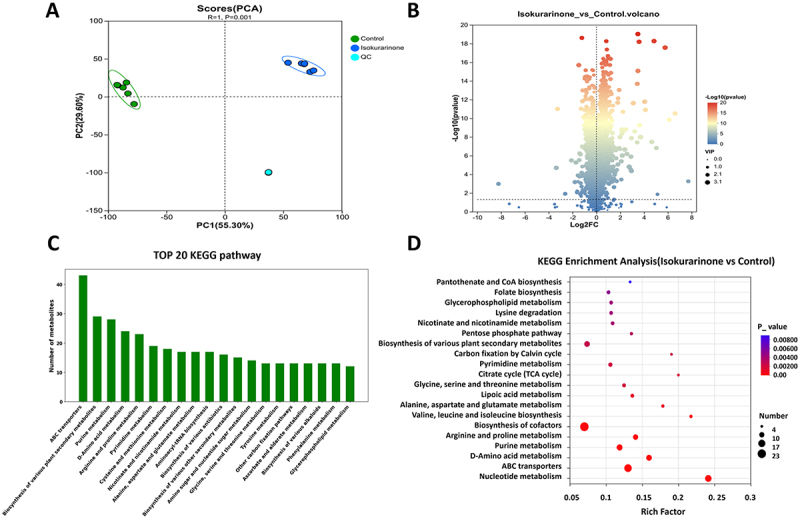
Metabolome analysis in MRSA after isokurarinone treatment. (A) PCA score plot of normalized LC/MS data from the control and isokurarinone groups (*n* = 6). (B) Volcano plot of differential metabolites; upregulated and downregulated metabolites are shown in red and blue, respectively. (C) numbers of differential metabolites in the top 20 KEGG pathways. (D) KEGG pathway enrichment analysis.

In the present study, we employed a metabolomics approach utilizing the UPLC-QTOF/MS system alongside multivariate statistical analysis to investigate the perturbation of the metabolite spectrum in MRSA treated with isokurarinone. Principal component analysis indicated that both control and isokurarinone treatment groups exhibited distinct clustering behavior, with significant differences observed in the metabolic profiles between the blank control group and the isokurarinone treatment group (Figure 5(A)). Specifically, 1207 metabolites were upregulated, while 636 metabolites were downregulated (Figure 5(B)). KEGG pathway enrichment analysis revealed that these differentiated metabolites (DMs) are involved in several pathways, including ABC transporters, purine metabolism, biosynthesis of cofactors, arginine and proline metabolism, D-amino acid metabolism, and pyrimidine metabolism (Figure 5(C,D)) [28]. Notably, the distinct alterations in pyrimidine metabolites, such as orotic acid, thymine, and cytidine diphosphate, suggest that isokurarinone may disrupt pyrimidine metabolism. Pyrimidine metabolism is a critical biochemical hub in bacteria, supplying essential nucleotides (UMP, CMP, TMP) necessary for nucleic acid synthesis, thereby supporting genetic replication and expression [38,39]. This pathway is intrinsically linked to cellular energy metabolism and membrane biogenesis through CTP-mediated phospholipid synthesis [40], thus sustaining key physiological processes required for bacterial growth. Its central role in coordinating genetic, energetic, and structural functions positions pyrimidine metabolism as a compelling multi-target avenue for the development of novel antibacterial strategies with potential applications in food safety and agricultural systems.

**Figure 6.**
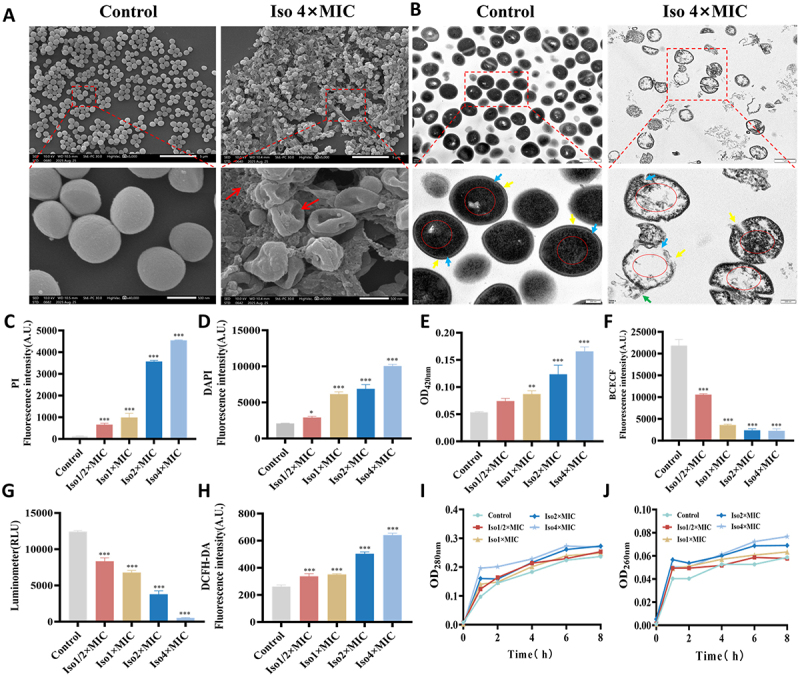
Isokurarinone exerts antibacterial activity by disrupting the bacterial membrane. (A) SEM images of MRSA after isokurarinone treatment. (B) TEM images of treated MRSA. (C–E) Membrane permeability assessed by PI, DAPI, and ONPG assays. (F) Proton motive force. (G) Intracellular ATP level. (H) ROS accumulation in MRSA after treatment. (I) Pleak of MRSA treated with isokurarinone. (J) Nucleic acid leak of MRSA treated with isokurarinone. Data are presented as mean ± SD (*n* = 3). Statistical analysis was performed using one-way ANOVA(*p < 0.05, **p < 0.01, ***p < 0.001).

The integrity and function of the inner membrane are essential for bacterial growth and survival [27]. To elucidate the mechanisms of isokurarinone, we examined the bacterial ultrastructure and morphology in MRSA following isokurarinone treatment using SEM and transmission electron microscopy (TEM) (Figure 6(A,B)). Compared to the control group, the bacteria exhibited significant morphological alterations, including severe irregularities in shape, cell wall rupture, and membrane damage, accompanied by a notable decrease in electron density and leakage of cellular contents after treatment with isokurarinone. As illustrated in Figure 6(C,D), the results demonstrated a significant increase in propidium iodide (PI) and 4,’6-diamidino-2-phenylindole (DAPI) fluorescence with increasing concentrations of isokurarinone, indicating that isokurarinone disrupts bacterial membrane integrity. Furthermore, the increased entry of ortho-nitrophenyl-β-galactoside (ONPG) into MRSA provided further insights into membrane destruction (Figure 6(E)). Moreover, we quantified the ΔpH using the fluorescent probe BCECF-AM to evaluate this critical component of the proton motive force (PMF). Our findings demonstrated that isokurarinone significantly disrupts the PMF compared to the control group (*p* < 0.001, Figure 6(F)). Given that PMF acts as the driving force for ATP synthesis, we observed a substantial reduction in intracellular ATP levels in MRSA (*p* < 0.001, Figure 6(G)). The disruption of membrane homeostasis leads to the accumulation of reactive oxygen species (ROS). In line with many bactericidal antibiotics, isokurarinone also induced ROS overproduction in a dose-dependent manner (*p* < 0.001, Figure 6(H)). To further determine whether isokurarinone-induced membrane disruption was accompanied by intracellular content leakage, the absorbance of bacterial supernatants at 280 nm and 260 nm was measured. As shown in Figure 6I,J, isokurarinone treatment caused a concentration-dependent increase in absorbance at both 260 nm and 280 nm compared with the untreated control. These results suggest that isokurarinone disrupted bacterial membrane integrity and promoted the leakage of proteins and nucleic acid from MRSA.

### Biocompatibility, antibiofilm activity, and virulence-related effects of isokurarinone

**Figure 7.**
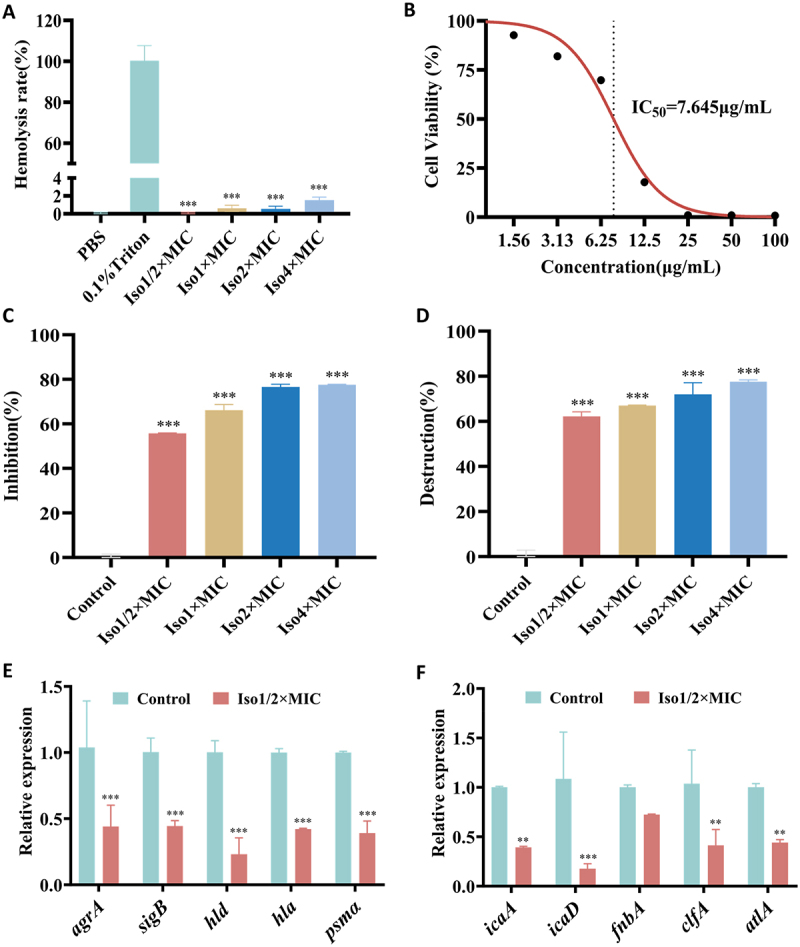
Effects of isokurarinone on biocompatibility, biofilm formation, and virulence in MRSA. (A) Hemolysis assay. (B) Cytotoxicity of isokurarinone for RAW 264.7 cells. (C) Inhibition of biofilm formation. (D) Disruption of mature biofilms. (E) Relative expression of virulence-associated and regulatory genes. (F) Relative expression of biofilm-associated genes. Data are presented as mean ± SD (*n* = 3). Statistical analysis was performed using one-way ANOVA (**p < 0.01, ***p < 0.001).

To assess host membrane compatibility, isokurarinone was directly co-incubated with sheep erythrocytes. No visible hemolysis was observed, even at 4 × MIC, indicating that there was no detectable hemolytic activity under the tested conditions (Figure 7(A)). To further evaluate the cellular safety of isokurarinone, a CCK-8 assay was performed using RAW264.7 cells. The result showed that isokurarinone reduced RAW264.7 cell viability in a concentration-dependent manner, with an IC_50_ value of 7.645 μg/mL (Figure 7(B)). Considering that the MIC of isokurarinone against MRSA was 4 μg/mL, the selectivity index against RAW264.7 cells was approximately 1.91, indicating a relatively narrow safety window in this macrophage-like cell line.

Given the central role of biofilms in the persistence of MRSA and treatment failure, we first evaluated the anti-biofilm activity of isokurarinone across a range of concentrations. Bacterial biofilms present a significant therapeutic challenge due to their matrix barrier, which limits antimicrobial penetration and promotes tolerance [41]. Consistent with its strong anti-MRSA activity, crystal violet staining revealed a clear concentration-dependent effect that isokurarinone significantly inhibited biofilm formation and disrupted pre-formed mature biofilms (Figure 7(C,D)).

To link these phenotypes to virulence regulation, we conducted RT-qPCR on planktonic MRSA exposed to 1/2 × MIC at selected time points to capture early regulatory shifts vs later biofilm remodeling. After 8 h, key virulence and regulatory markers (*agrA*, *sigB*, *hld [RNAIII]*) and toxin-associated genes (*hla*, *psmα*) were significantly downregulated compared to controls (*p* < 0.001, Figure 7(E)). After 24 h, biofilm and adhesion determinants were also diminished *icaA*, *clfA*, and *atlA* showed significant reductions (*p* < 0.01), while *icaD* was even more strongly suppressed (*p* < 0.001). In contrast, *fnbA* exhibited no significant change under the same conditions (Figure 7(F)). Collectively, these data indicate that sub-MIC isokurarinone is associated with the early suppression of toxin-linked programs, followed by a broader downregulation of biofilm-associated transcription, which aligns with its inhibitory and disruptive effects on MRSA biofilms.

### Acute oral toxicity assessment of isokurarinone

**Figure 8.**
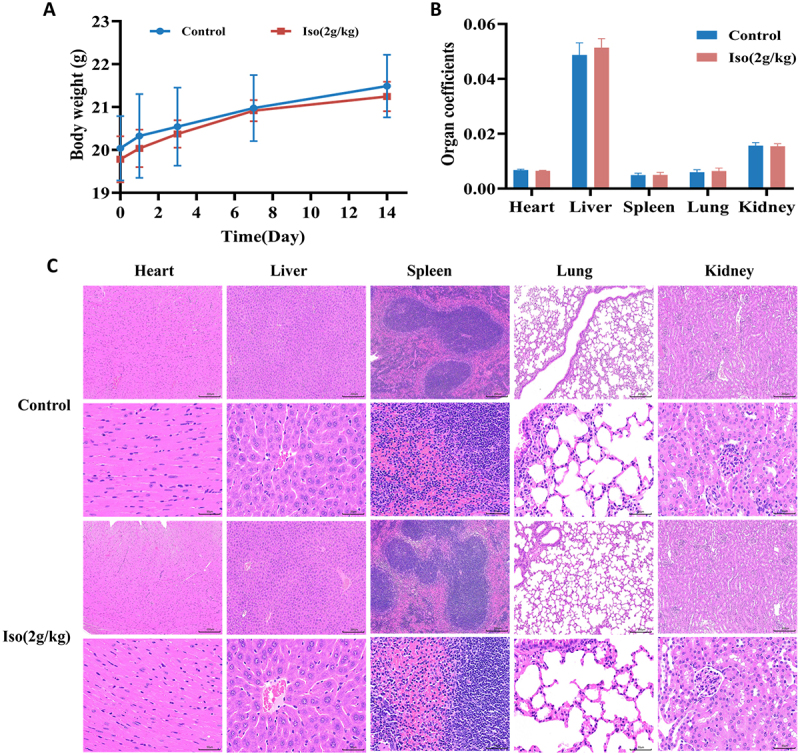
Acute oral toxicity evaluation of isokurarinone in mice. (A) Changes in body weight. (B) Organ coefficients of major organs. (C) Representative HE staining images of major organs.

In the acute oral toxicity assay, mice were given a single oral dose of isokurarinone at 2 g/kg and monitored for 14 days. No mortality or overt toxic signs were observed. Body weight increased steadily (Figure 8(A)), and no significant changes were detected in the organ coefficients of the heart, liver, spleen, lung, or kidney (Figure 8(B)). Hematological and serum biochemical parameters remained within normal physiological ranges (Figure S4). H&E staining showed no evident pathological lesions in the major organs (Figure 8(C)). These findings suggest that isokurarinone was well tolerated at a single oral dose of 2 g/kg under the present experimental conditions.

### Therapeutic effect of isokurarinone on MRSA-infected wound model in mice

As the body’s primary line of defense, the skin is continuously exposed to environmental insults. Severe injuries resulting from trauma, burns, or disease pose significant clinical challenges, often complicated by secondary bacterial infections. Among these infections, Methicillin-resistant *Staphylococcus aureus* (MRSA) is particularly prevalent, frequently inducing chronic inflammation that markedly hinders the transition of wound healing from the inflammatory phase to the proliferation and remodeling phases [28]. Concurrently, research indicates that bacterial biofilm formation alters the wound microenvironment, exhibiting strong resistance to both antibiotics and host defense mechanisms, thereby posing a considerable barrier to chronic wound healing [42].

**Figure 9.**
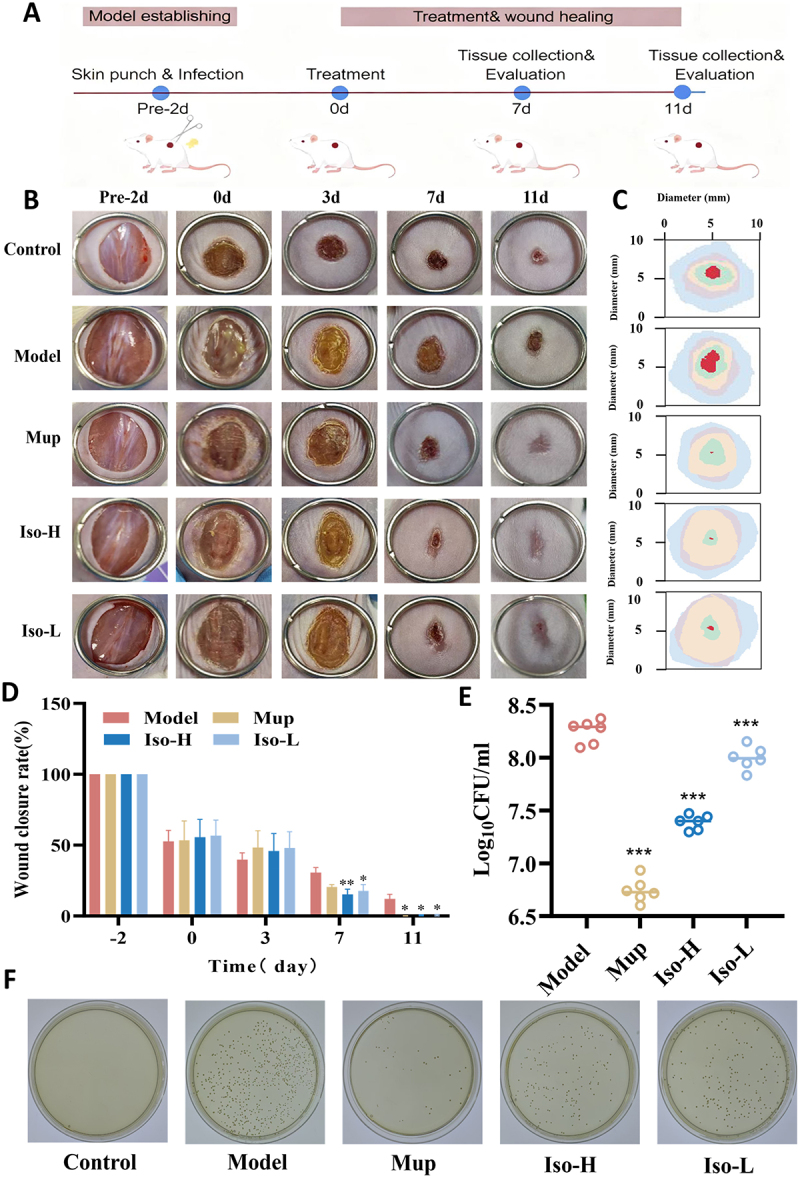
Isokurarinone promoted MRSA-infected wound healing. (A) Schematic of the animal experiment. (B) Representative photographs of infected full-thickness wounds. (C) Tracings of wound closure over 11 days. (D) Quantification of wound closure rate (*n* = 6). (E) Bacterial counts in wound tissues on day 7 (*n* = 6). (F) Representative images of wound exudates on day 7. Data are presented as mean ± SD. Statistical analysis was performed using one-way or two-way ANOVA (*p < 0.05, **p < 0.01, ***p < 0.001).

To further investigate whether isokurarinone promotes healing of wounds infected with MRSA, a full-thickness skin-wound model was established. The wounds were observed on days 0, 3, 7, and 11. Subsequently, wound tissue was collected on days 7 and 11 for analysis (Figure 9(A)). Macroscopic evaluation revealed superior wound healing in isokurarinone-treated mice on days 7 and 11 compared to the other groups (Figure 9(B)). The progression of wound healing over the 11-day treatment period was recorded using various colors (Figure 9(C)). As illustrated in Figure 9(D), on day 7, the unhealed wound areas were 30.69 % and 20.45 % in mice treated with PBS and Mup, respectively, while 15.34 % and 17.76 % (*p* < 0.01 or *p* < 0.05) of the wound area remained after treatment with Iso-H and Iso-L. On day 11, the uncovered wound area in PBS-treated mice accounted for 12.13 %, which was significantly greater than those in the Mup (0.12 %, *p* < 0.05), Iso-H (0.42 %, *p* < 0.05), and Iso-L (0.55 %, *p* < 0.05) groups.

Moreover, the bacterial loads in wound skin were significantly reduced after treatment with Iso-H and Iso-L compared to the PBS-treated group on day 7 (*p* < 0.001, Figure 9(E)). Representative application plates of wound site secretions on day 7 are shown in Figure 9(F), demonstrating the effective elimination of wound bacteria by isokurarinone treatment. These results indicate a greater wound-healing efficacy than that of Mupirocin.

**Figure 10.**
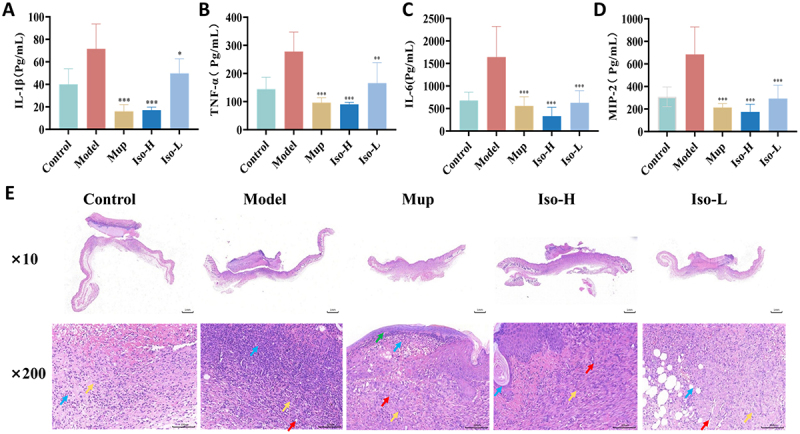
Inflammatory evaluation and histological of MRSA-infected wound tissues on day 7. (A-D) Levels of IL-1β, TNF-α, IL-6, and MIP-2 in wound tissues on day 7, measured by ELISA (*n* = 6). (E) H&E staining of wound tissues on day 7. Blue arrows indicate inflammatory cell infiltration, red arrows indicate neovascularization, and yellow arrows indicate fibroblast deposition. Data are presented as mean ± SD. Statistical analysis was performed using one-way ANOVA (*p < 0.05, **p < 0.01, ***p < 0.001).

Anti-inflammatory activity is crucial in the wound healing process. During bacterial clearance, the excessive release of inflammatory factors disrupts the balance with growth factors, inhibiting cell proliferation and ultimately delaying wound healing. Consequently, the degree of inflammatory resolution correlates positively with the advancement of wound repair [43]. Therefore, therapeutic strategies aimed at mitigating inflammatory mediators represent a viable approach to facilitating the resolution of inflammation and promoting effective tissue repair. The levels of cytokines and chemokines in wound tissues after 7 days of treatment were assessed using ELISA. IL-1β, TNF-α, and IL-6 are key early inflammatory mediators. As shown in Figure 10(A–C), treatment with isokurarinone, particularly in the Iso-H group, resulted in significant reductions in the levels of these cytokines. Chemokines play a critical role in coordinating the transition from inflammation to proliferation during wound healing [44]. Consistently, isokurarinone treatment significantly reduced the level of the key chemokine MIP-2 compared to the model group (*p* < 0.001, Figure 10(D)), indicating a facilitated progression into the proliferative phase. The histopathological evaluation conducted on day 7 revealed the formation of granulation tissue and extensive infiltration of inflammatory cells in both the Control and Model groups, characterized by disordered fibroblast arrangement. In contrast, the positive drug and isokurarinone groups also displayed granulation and significant inflammation; however, the fibers began to align in a more orderly pattern (Figure 10(E)).

**Figure 11.**
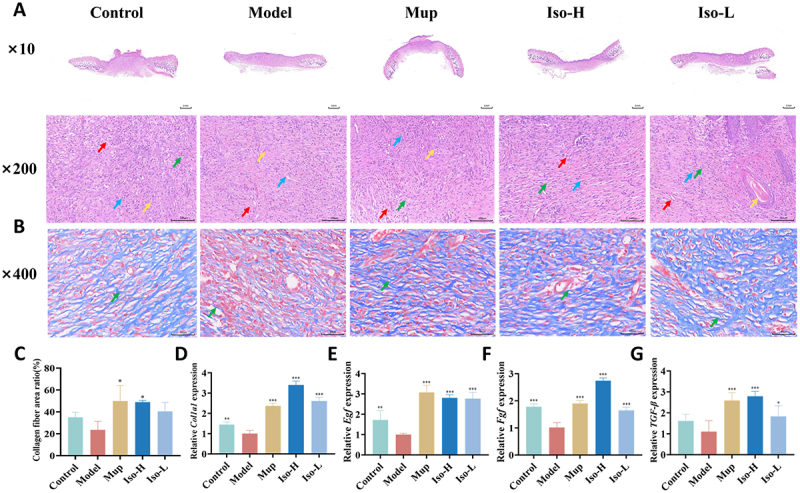
Wound remodeling and repair responses in MRSA-infected tissues on day 11. (A) Representative H&E staining images. Blue arrows indicate inflammatory cell infiltration, red arrows indicate neovascularization, and yellow arrows indicate fibroblast deposition. (B) Masson trichrome staining; green arrows indicate collagen fibers. (C) Quantification of collagen deposition (*n* = 3). (D-G) Relative expression of *Col1a1, Egf, Fgf*, and *Tgf-β* in wound tissues. Data are presented as mean ± SD. Statistical analysis was performed using one-way ANOVA (*p < 0.05, **p < 0.01, ***p < 0.001).

By day 11, the isokurarinone-treated group exhibited a restored skin structure, marked by orderly arranged subcutaneous fibrous tissue and a reduction in inflammatory cell infiltration (Figure 11(A)). Masson trichrome staining was performed to assess newly formed collagen across the various treatment groups (Figure 11(B)). The collagen deposition ratios in the Mup and Iso-H groups were 49.97 % and 48.92 %, respectively, which were significantly higher than the model group’s ratio of 23.51 % (*p* < 0.05, Figure 11(C)). These findings indicate that isokurarinone effectively promotes collagen synthesis and deposition during the wound healing process.

Next, a systematic evaluation of angiogenesis and epithelialization in the wounds was conducted using RT-qPCR. The factors involved in angiogenesis and epithelialization collaboratively promote cell proliferation, vascular formation, and collagen deposition, all of which are essential for wound healing and skin repair. As indicated in Figure 11(D), the expression of *Col1a1* in the treatment group was significantly increased (*p < 0.001*). Based on the results from Masson trichrome staining, which demonstrated collagen deposition, we propose that isokurarinone not only activates the molecular program in fibroblasts to synthesize collagen but also effectively translates this synthetic activity into extracellular matrix deposition. Consistently, as shown in Figures 11(E–G), treatment with isokurarinone led to a significant upregulation of the expression levels of *Egf*, *Fgf*, and *TGF-β* on day 11, compared to the Model group [32,45]. This upregulation of these genes strongly indicates the activation of the endogenous repair program, facilitating the transition of the wound from the inflammatory phase to the subsequent proliferative and remodeling phases.

## Discussion

The management of methicillin-resistant *Staphylococcus aureus* (MRSA) remains a formidable global health challenge due to its multifactorial antimicrobial resistance [46], which compromises the efficacy of many conventional antibiotics, and increases reliance on last-line agents [47,48]. This ongoing pressure highlights the urgent need for therapeutics with novel mechanisms and strategies that diminish persistence-associated traits that promote chronic infection. Natural products continue to serve as privileged chemical scaffolds for antibiotic discovery and mechanistic probes, offering structural diversity that can uncover exploitable bacterial vulnerabilities [49,50]. Among these, prenylated flavonoids have consistently demonstrated activity against MRSA, and the process of prenylation can enhance antibacterial potency, rendering these scaffolds appealing starting points for further optimization [51].

In this study, we employed a target-guided discovery strategy focused on aspartate transcarbamoylase (ATCase), which catalyzes the initial committed step of *de novo* pyrimidine biosynthesis. Screening a flavonoid natural product library identified isokurarinone as a lead candidate, demonstrating both favorable in silico ranking and potent *in vitro* anti-MRSA activity. Notably, the observed antibacterial activity was accompanied by a reduction in ATCase enzymatic activity and a decrease in *pyrB* expression, thereby supporting the engagement of the intended metabolic node within the cellular context.

Multiple, orthogonal lines of evidence support a direct interaction between isokurarinone and ATCase. Docking studies suggested a plausible binding mode involving hydrogen bonding and hydrophobic contacts with key residues (THR49, ARG50, SER159, VAL161, ARG210), while molecular dynamics simulations indicated the formation of a stable complex. In agreement with these predictions, surface plasmon resonance (SPR) demonstrated micromolar-affinity binding (K_D_ = 2.45 μM), and differential scanning fluorimetry (DSF) showed ligand-dependent thermal stabilization of ATCase. Collectively, these computational and biophysical data support the notion that ATCase is a key target engaged by isokurarinone in MRSA.

Previous studies on inhibitors of nucleotide biosynthesis have shown that disruption of pyrimidine or purine metabolism can impair bacterial growth, reduce metabolic adaptability, and sensitize bacteria to additional cellular stress [10]. Although most reported anti-MRSA agents clinically used today, such as vancomycin and mupirocin, primarily interfere with cell-wall biosynthesis or protein synthesis, respectively, targeting ATCase represents a distinct metabolic strategy by acting upstream of pyrimidine nucleotide availability. Similar to other target-guided antibacterial discovery studies, our work combined structure-based screening, molecular dynamics simulation, enzymatic activity measurement, and biophysical binding validation [52,53]. Compared with studies relying only on docking prediction, the additional SPR and DSF evidence strengthens the conclusion that isokurarinone directly engages ATCase. Therefore, the present findings place isokurarinone among metabolism-oriented antibacterial candidates and suggest that ATCase-associated pyrimidine biosynthesis may represent an exploitable vulnerability in MRSA.

Consistent with ATCase engagement, non-targeted metabolomics revealed significant perturbations in pyrimidine-related metabolism. A mechanistic link between pyrimidine disequilibrium and envelope damage is biologically plausible, as pyrimidine nucleotides are essential for activating precursors of major cell-envelope constituents, including peptidoglycan, teichoic acids, and phospholipids [8]. For instance, CTP-dependent cytidylyltransferases generate CDP-glycerol, an activated precursor utilized in the biogenesis of Gram-positive teichoic acid and linkage units. Thus, pyrimidine limitation may restrict teichoic acid assembly, compromising the stability of the cell-wall-associated envelope [54]. Concurrently, CTP is also necessary for synthesizing CDP-diacylglycerol, a central liponucleotide intermediate in bacterial phospholipid synthesis, thereby linking pyrimidine availability directly to membrane lipid homeostasis [55]. Consequently, pyrimidine starvation has been proposed to diminish the availability of UDP-sugars required for cell-wall biosynthesis and to interact with envelope stress responses. This provides an additional pathway through which nucleotide limitation can sensitize the cell surface to damage and impair repair capacity once injury occurs [7].

Guided by these findings, we identified pronounced membrane injury through SEM/TEM analysis, increased permeability via PI/DAPI staining and ONPG hydrolysis, and subsequent energetic stress characterized by a reduced proton motive force and ATP depletion [56,57]. These changes were accompanied by reactive oxygen species (ROS) accumulation. The concentration-dependent increases in OD_280_ and OD_260_ further support the membrane-disruptive effect of isokurarinone. Combined with membrane permeability assays, ATP depletion, ROS accumulation, and SEM/TEM observations, this finding suggests that isokurarinone impairs bacterial envelope integrity and disrupts intracellular homeostasis.

These phenotypic changes are in line with antibacterial mechanisms reported for other natural products and small-molecule inhibitors investigated using similar experimental approaches [58–60]. Studies employing PI uptake, DAPI staining, ONPG hydrolysis, ATP measurement, ROS detection, and electron microscopy have frequently linked antibacterial activity to increased membrane permeability, membrane potential disruption, intracellular energy depletion, oxidative stress, and ultrastructural damage. Although isokurarinone shares several downstream phenotypes with membrane- and metabolism-disrupting compounds, the present data do not establish whether it directly interacts with specific membrane-surface components. Based on the ATCase-targeting evidence and metabolomic results, the observed membrane disruption may be secondary, at least in part, to ATCase-associated pyrimidine metabolic disturbance. Pyrimidine nucleotides are required for the biosynthesis of envelope-related precursors. Thus, their impaired availability may weaken envelope maintenance and repair and increase membrane vulnerability. Therefore, isokurarinone may exert its anti-MRSA activity through a cascade involving pyrimidine metabolic disturbance, impaired envelope maintenance, membrane permeability changes, energetic collapse, ROS accumulation, and intracellular contents leakage, rather than through a single isolated effect. Further lipidomic profiling, cell-wall precursor analysis, membrane protein interaction assays, and rescue experiments are needed to determine whether isokurarinone directly targets specific membrane components or indirectly induces membrane disruption through metabolic imbalance.

A key aspect of virulence is the mechanistic coupling between bacterial physiology and virulence outputs that drive host damage and persistence [61]. Our phenotypic data support a virulence-relevant interpretation. Isokurarinone demonstrated strong activity against both planktonic cells and biofilm-associated populations, providing functional evidence for the coordinated repression of biofilm- and regulator-associated markers. Additionally, a standard erythrocyte hemolysis assay revealed no detectable hemolytic activity toward sheep red blood cells, with no visible hemolysis observed even at 4 × MIC. This finding offers a preliminary selectivity signal: despite inducing membrane and energetic stress in bacteria, isokurarinone did not exhibit overt membrane-disruptive toxicity toward erythrocytes under the tested conditions. However, hemolysis represents only one dimension of host compatibility and should be complemented by broader cytotoxicity and tolerability profiling [62]. The RAW264.7 cytotoxicity assay indicated concentration-dependent cytotoxicity, suggesting that the current safety profile of isokurarinone remains preliminary and that its therapeutic window should be further optimized. Future studies should include broader cytotoxicity testing, as well as repeated-dose toxicity, local dermal safety evaluation, pharmacokinetic characterization, and formulation optimization.

A central theme in staphylococcal pathogenesis is the mechanistic coupling of virulence outputs to the bacterial physiological state through integrated regulatory networks that sense stress and metabolic cues, subsequently reprogramming the expression of adhesins, biofilm determinants, and toxins [63]. In this context, RT-qPCR data provide a mechanistic link between physiological perturbations and virulence-relevant outputs. Under 1/2 × MIC isokurarinone exposure, the early repression (8 h) of the agr axis (*agrA* and its *RNAIII/hld* readout), along with a reduction in *hla* and *psmα*, supports the attenuation of toxin-linked programs that contribute to host damage and immune modulation, consistent with the established role of *Agr/RNAIII* in coordinating the expression of secreted toxins [64]. Notably, the concomitant reduction in *sigB* aligns with broader regulatory remodeling under sub-inhibitory stress [65].

At 24 h, the downregulation of *icaA* and *icaD* suggests a reduced capacity for the production of PIA/PNAG-dependent matrix. Meanwhile, the decreased expression of *clfA* and *atlA* is consistent with weakened adhesion and altered cell-surface remodeling, which contribute to attachment, biofilm architecture, and the stability of the eDNA-associated matrix [66]. The lack of a significant change in *fnbA*, despite the broad suppression of other adhesion and matrix-associated genes, likely reflects a gene-specific regulatory architecture and timing [67]. Taken together, the overall pattern indicates an early reduction in *Agr* and *RNAIII*-associated toxin programs, followed by a decrease in the expression of core matrix and adhesion determinants. This supports the hypothesis that isokurarinone contributes to anti-virulence at sub-MIC levels and complements its antibacterial activity by limiting toxin-mediated damage and reducing biofilm-associated persistence.

The acute oral toxicity results further support the preliminary *in vivo* tolerability of isokurarinone. No mortality, abnormal body weight loss, hematological or serum biochemical abnormalities, or histopathological lesions in major organs were observed after a single oral dose of 2 g/kg. These findings suggest that isokurarinone did not cause obvious acute systemic toxicity under the tested conditions. Nevertheless, the safety interpretation remains preliminary. Acute oral toxicity only reflects short-term tolerance after single-dose exposure and cannot replace repeated-dose, local dermal, or pharmacokinetic safety evaluations.

The therapeutic relevance of the combined antibacterial and virulence-linked effects was demonstrated *in vivo* using a MRSA-infected wound model. Isokurarinone accelerated wound closure and reduced bacterial burden, showing favorable results compared to mupirocin in this context. Additionally, treatment was associated with a decrease in local inflammatory mediators, which aligns with improved pathogen clearance and a wound microenvironment that is more conducive to repair. Correspondingly, enhanced collagen deposition and increased expression of pro-repair growth factors indicate a better progression toward re-epithelialization and tissue remodeling. While these host responses are likely driven primarily by bacterial clearance, the potential direct contribution of isokurarinone to immunomodulation cannot be ruled out and deserves further investigation.

Several limitations should be acknowledged. Although multiple lines of evidence support ATCase as a potential target of isokurarinone, the current data cannot fully establish ATCase as the primary cellular target in MRSA. Virtual screening, molecular dynamics simulations, SPR, and DSF support a plausible and direct interaction, but they do not exclude the possibility of additional targets or secondary antibacterial stress responses. Definitive target-function validation will require genetic and rescue experiments, including *pyrB* overexpression or complementation, target-site mutagenesis of predicted binding residues, pyrimidine supplementation assays, and affinity-based target profiling. In addition, although the *in vitro* combination assay provides preliminary information on the interaction between isokurarinone and conventional anti-MRSA antibiotics, further *in vivo* combination therapy studies are needed to determine whether such interactions can translate into improved bacterial clearance, wound healing, inflammatory control, and safety in infected animals. The advancement of isokurarinone will also require medicinal chemistry optimization, pharmacokinetic characterization, repeated-dose and local safety evaluations, and broader toxicity profiling before its therapeutic potential can be fully established.

## Conclusion

In summary, we identify isokurarinone as a natural product that engages with ATCase and exhibits potent anti-MRSA activity both *in vitro* and *in vivo*. By integrating target engagement, metabolomic remodeling, virulence-associated gene changes, and efficacy in infection models, our findings support ATCase as a viable metabolic vulnerability in MRSA. Furthermore, this work provides a foundation for the mechanism-guided development of targeted therapeutics against MRSA wound infections.

## Supplementary Material

Supplemental Material

## Data Availability

The data that support the findings of this study are directly available in 4TU. ResearchData at https://data.4tu.nl/datasets/4a8bc3f3-ffe7-4ab2-8f42-95cdf65d1151/1 [68]. The metabolome data were available in the Metabolights repository (accession code: MTBLS13673; https://www.ebi.ac.uk/metabolights/editor/MTBLS13673/descriptors).
