# Supplementary material for: Discovery of isokurarinone as an ATCase-engaging lead with potent activity against methicillin-resistant Staphylococcus aureus
Source: Virulence. 2026 Jul 21;17(1):2707766. doi: 10.1080/21505594.2026.2707766 (PMC13418473; doi:10.1080/21505594.2026.2707766)
Supplement: Supplemental Material [file KVIR_A_2707766_SM5653.docx]

**Supplementary Material**

**Discovery of isokurarinone as an ATCase-engaging lead with potent activity against methicillin-resistant *Staphylococcus aureus***

Xinyuan Cao^a^†, Xiaorong Yang^b^†, Lixia Dai^b^, Xueyan Liu^b^, Zile Gong^b^, Xiaoyan Yu^b^, Yuchao Ma^b^, Tong Bu^b^, Haowen Wu^b^, Xiaolou Miao^b^, Xiaofei Shang^b*^, Changcai Bai^a*^

^a^ School of Pharmacy, Ningxia Medical University, Yinchuan 750004, China;

^b^ Key Laboratory of Veterinary Pharmaceutical Development of Ministry of Agriculture, Key Laboratory of New Animal Drug Project, Gansu Province, Lanzhou Institute of Husbandry and Pharmaceutical Sciences, Chinese Academy of Agricultural Sciences, Lanzhou 730050, China.

†These authors contributed equally to this work.

^*^To whom correspondence should be addressed.

Email: changcaibai@163.com; shangxf928@126.com

**Content**

**Table S1** Strains used in this study

**Table S2** Primers used in this study

**Figure S1** RAMACHANDRAN analysis

**Figure S2** PCA and FEL analysis

**Figure S3** Checkerboard assay of isokurarinone combined with vancomycin against MRSA

**Figure S4** Effects of isokurarinone on blood parameters. (A) Routine blood parameters. (B) Serum biochemical parameters

**Table S1** Strains used in this study

| **Strains** | **Description** | **Reference /source** |
| --- | --- | --- |
| *Staphylococcus aureus* ATCC43300 | Standard strain | ATCC |
| *Staphylococcus aureus* ATCC29213 | Standard strain | ATCC |
| *Enterococcus faecalis* ATCC29212 | Standard strain | ATCC |
| *Escherichia coli* ATCC25922 | Standard strain | ATCC |
| *Proteus vulgaris* ATCC49132 | Standard strain | ATCC |
| *Pseudomonas aeruginosa* ATCC27318 | Standard strain | ATCC |
| *Salmonella typhimurium* ATCC14028 | Standard strain | ATCC |

**Table S2** Primers used in this study

| **Primer** | **Oligonucleotide (5′-3′)** | **Application** |
| --- | --- | --- |
| RT-*pyrB*-F | AGACATGGGCTTGCAGAAGA | qRT-PCR |
| RT-*pyrB*-R | CCTCTATTCACAGGTGCCGG | qRT-PCR |
| RT-*agrA*-F | GCTTTGTCGTCAATCGCCATA | qRT-PCR |
| RT-*agrA*-R | TCTCACCGATGCATAGCAGT | qRT-PCR |
| RT-*sigB*-F | GGTGCCATAAATAGATTCGATATGTCCTT | qRT-PCR |
| RT-*sigB*-R | CTTTTGATTTCACCGATTACAGTAGGTACT | qRT-PCR |
| RT-*hld*-F | AGGAAGGAGTGATTTCAATGGC | qRT-PCR |
| RT-*hld*-R | GTGAATTTGTTCACTGTGTCGAT | qRT-PCR |
| RT-*hla*-F | TTGGAACCCGGTATATGGCA | qRT-PCR |
| RT-*hla*-R | ACTGTAGCGAAGTCTGGTGA | qRT-PCR |
| RT-*psmα*-F | GGCCATTCACATGGAATTCGTAG | qRT-PCR |
| RT-*psmα*-R | TAGCCATCGTTTTGTCCTCCTG | qRT-PCR |
| RT-*icaA*-F | GTTGTCGACGTTGGCTACTG | qRT-PCR |
| RT-*icaA*-R | ATGGCAAGCGGTTCATACTT | qRT-PCR |
| RT-*icaD*-F | ACAATACGTGTTGCTTTAAACATTG | qRT-PCR |
| RT-*icaD*-R | CGATTCTCTTCCTCTCTGCCA | qRT-PCR |
| RT-*fnbA*-F | GCGCAGTGAGCGATCATACA | qRT-PCR |
| RT-*fnbA*-R | CTCGACTGGTCCTTGTGCTT | qRT-PCR |
| RT-*clfA*-F | TTGGCGCTCTATGTCATGGG | qRT-PCR |
| RT-*clfA*-R | CACCAGGCTCATCAGGTTGT | qRT-PCR |
| RT-*atlA*-F | AACAGCACCAACGGATTAC | qRT-PCR |
| RT-*atlA*-R | CATAGTCAGCATAGTTATTCATTG | qRT-PCR |
| RT-*16S rRNA*-F | CGCAATGGGCGAAAGC | qRT-PCR |
| RT-*16S rRNA*-R | TACGATCCGAAGACCTTCATCA | qRT-PCR |
| RT-*Egf*-F | GGACTGAGTTGCCCTGACTC | qRT-PCR |
| RT-*Egf*-R | CAATATGCATGCACACGCCA | qRT-PCR |
| RT-F*gf*-F | GGCTGCTGGCTTCTAAGTGT | qRT-PCR |
| RT-*Fgf*-R | GTCCCGTTTTGGATCCGAGT | qRT-PCR |
| RT-*Tgfβ*-F | ACTGGAGTTGTACGGCAGTG | qRT-PCR |
| RT-*Tgfβ*-R | GGGGCTGATCCCGTTGATTT | qRT-PCR |
| RT-*Col1a1*-F | TTCTCCTGGCAAAGACGGAC | qRT-PCR |
| RT-*Col1a1*-R | CGGCCACCATCTTGAGACTT | qRT-PCR |
| RT-*GAPDH*-F | GCTGAGTATGTCGTGGAGT | qRT-PCR |
| RT-*GAPDH*-R | GTTCACACCCATCACAAAC | qRT-PCR |


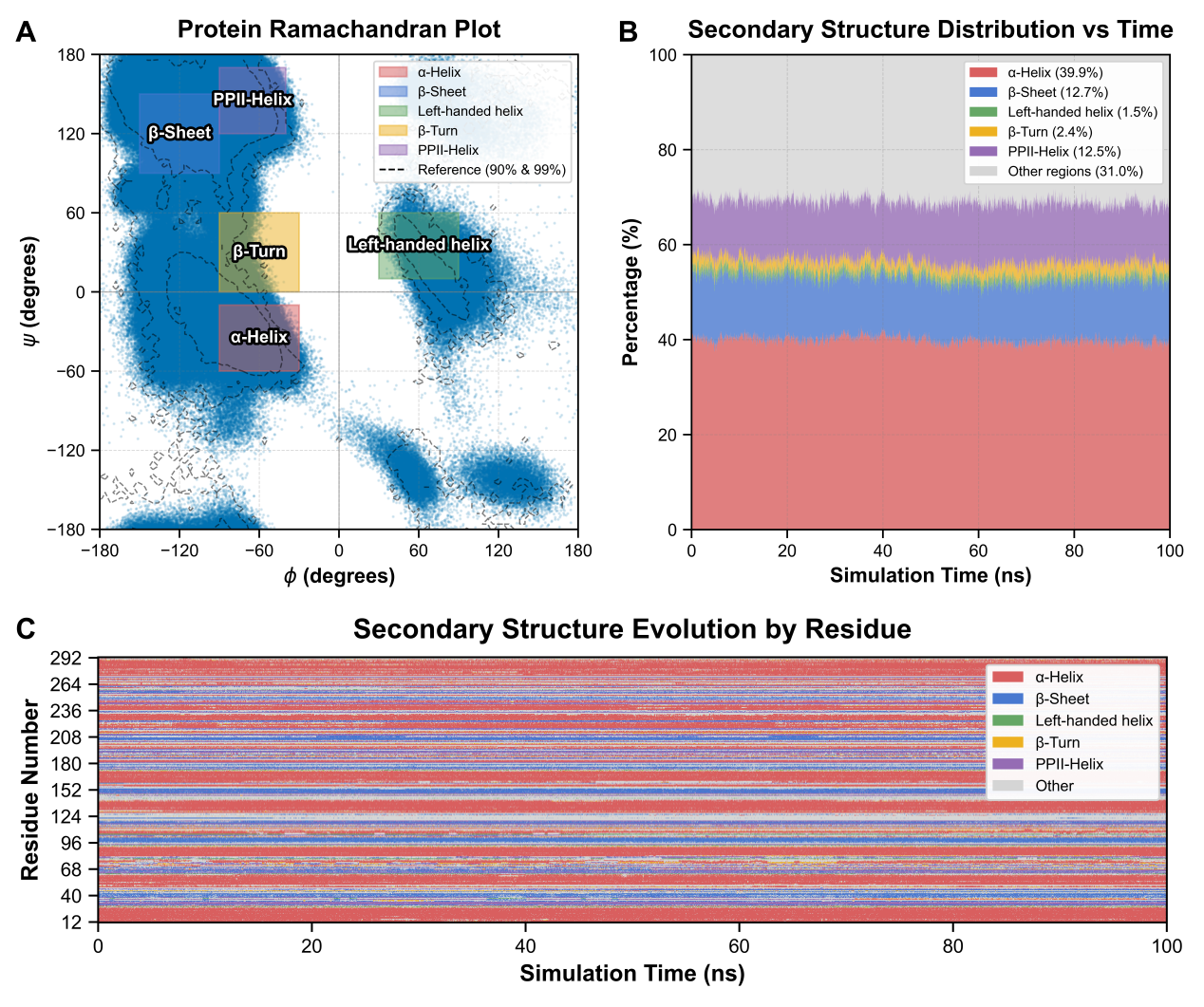


**Figure S1** RAMACHANDRAN analysis. (A) RAMACHANDRAN figure of ATCase. (B) Protein ramachandran plot. (C) Secondary structure distribution vs time.


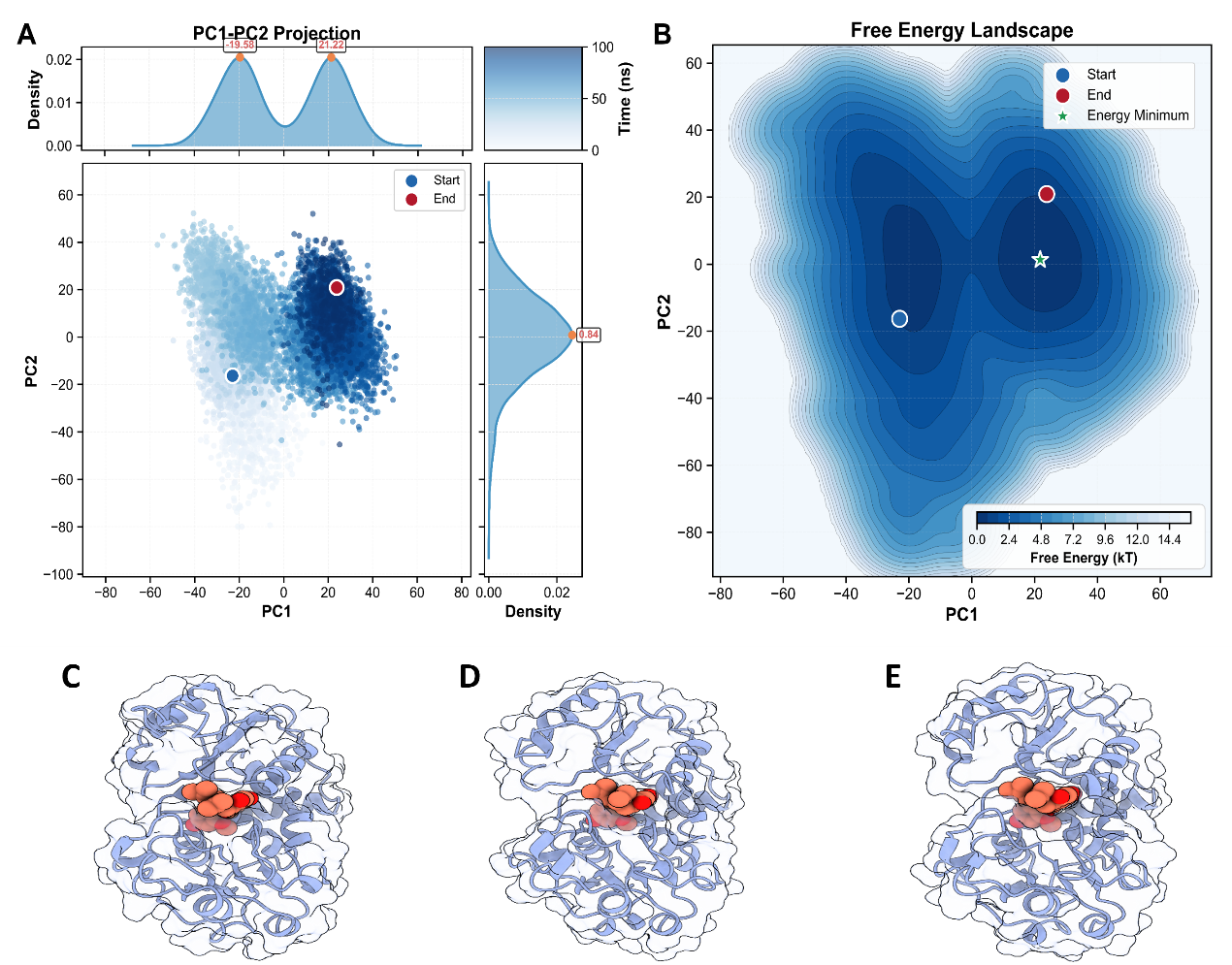


**Figure S2** PCA and FEL analysis. (A) Principal Component Analysis (PCA). (B) Free Energy Landscape. (C-E) Structures of the complexes of isokurarinone with ATCase at Start, End and Energy Minimum.

**Figure S3** Checkerboard assay of isokurarinone combined with vancomycin against MRSA.


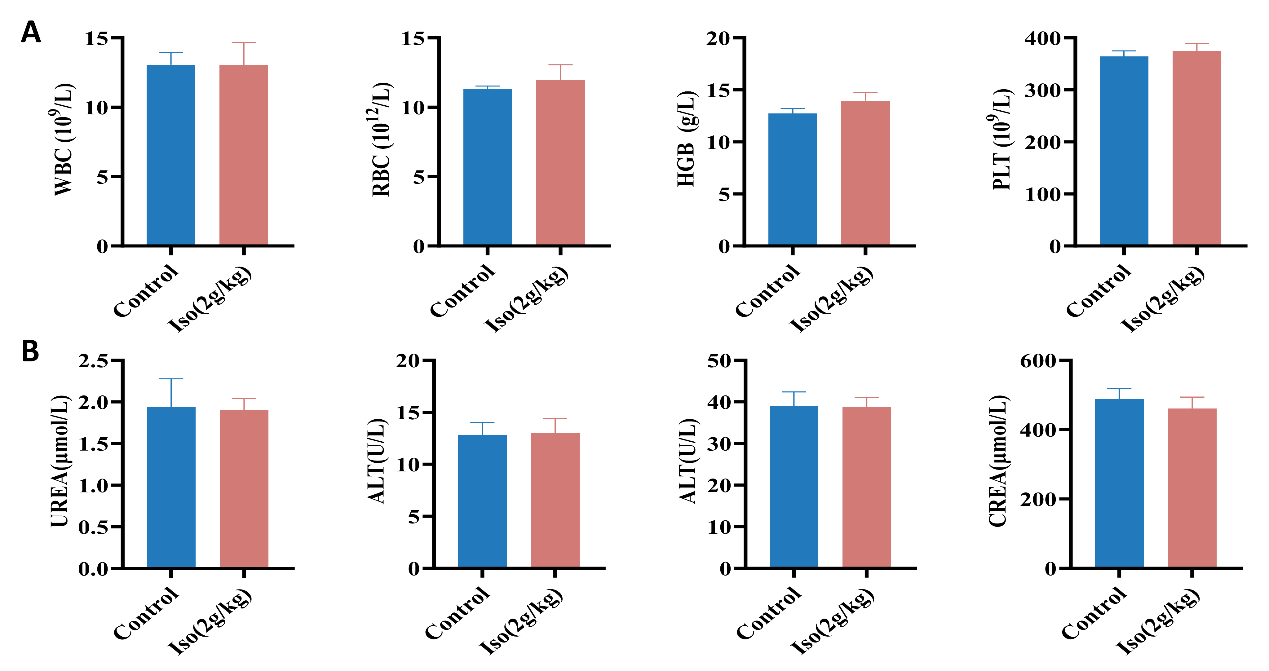


**Figure S4** Effects of isokurarinone on blood parameters. (A) Routine blood parameters. (B) Serum biochemical parameters.
